# Image analysis for cervical cancer classification using deep learning techniques

**DOI:** 10.1186/s12885-026-16076-1

**Published:** 2026-04-23

**Authors:** Emmanuel Christian Nyabyenda, Kizito Nkurikiyeyezu, Muzungu Hirwa Sylvain, Gashaija Absolomon, Isaac Komezusenge, Fauste Ndikumana, Melissa Uwase, Jean Damascene Hagenimana, Piero Mazimpaka Irakiza, Felix K. Rubuga, Dieudonne Kayiranga, Belson Rugwizangoga

**Affiliations:** 1 Centre for Impact, Innovation and Capacity Building for Health Information Systems and Nutrition, (CIIC-HIN), Kigali, Rwanda; 2 University of Rwanda, African Centre of Excellence in Data Science, Kigali, Rwanda; 3 African Centre of Excellence in Biomedical Engineering and e-Health, University of Rwanda, Kigali, Rwanda; 4 School of Public Health, University of Rwanda, Kigali, Rwanda; 5 School of Nursing and Midwifery, College of Medicine and Health Sciences, University of Rwanda, Kigali, Rwanda; 6 University Teaching Hospital of Kigali (CHUK), Kigali, Rwanda; 7 School of Medicine and Pharmacy, University of Rwanda, Kigali, Rwanda

**Keywords:** Histopathological images, Medical image analysis, Class imbalance, Binary classification, Multi-class classification

## Abstract

**Background:**

Cervical cancer is a leading cause of cancer-related molarity among women in sub-Sahara Africa, where limited pathology capacity constrains timely diagnosis. Rwanda reports one of the highest cervical cancers incidence rates in the region, and there is a pressing need for scalable decision-support tools to augment histopathology services. This study evaluated deep learning models for automated binary classification of cervical histopathology images from a tertiary hospital in Kigali, Rwanda.

**Methods:**

We conducted a retrospective, single-center study using 885 hematoxylin-and-eosin-stained cervical biopsy image patches acquired at the University Teaching Hospital of Kigali (CHUK) between 2018 and 2024. Regions of interest were annotated by board-certified pathologists and organized into normal versus abnormal (precancerous and malignant) categories. Three ImageNet-pretrained convolutional neural networks (ResNet50, EfficientNetB0, DenseNet121) were fine-tuned using patient-level, stratified splits (70% training, 20% validation, 10% test), class-weighted loss, and domain consistent data augmentation. Performance was assessed on the held-out test set using accuracy, sensitivity, specificity, F1-score, receiver operating characteristic area under the curve (ROC-AUC), Brier score, and calibration curves with 95% confidence intervals.

**Results:**

EfficientNetB0 achieved the best overall performance, with test accuracy 0.99 (95% CI 0.97-1.00), sensitivity 0.98, specificity 1.00, F1-score 0.99, ROC-AUC 0.99, and the lowest Brier score (0.02). ResNet50 reached an accuracy of 0.91 (ROC-AUC 0.96), while DenseNet121 obtained an accuracy of 0.86 (ROC-AUC 0.96), with comparatively poorer calibration. EfficientNetB0 misclassified only one abnormal image as normal and produced no false positives on the test set.

**Conclusions:**

Compact deep learning architectures, particularly EfficientNetB0, can deliver near-perfect discrimination between normal and abnormal cervical histopathology in a resource constrained Rwandan setting. These results support the feasibility of AI-assisted pathology as a triage tool but require external, multi-center and prospective validation before clinical deployment.

## Introduction

Cervical cancer is a leading cause of cancer-related deaths among women globally, with an estimated 350,000 deaths and 660,000 new cases reported in 2022, making it the fourth most diagnosed cancer worldwide among both sexes [1, 2]. The disease primarily results from persistent infection with high-risk human papillomavirus (HPV), which can lead to precancerous lesions and, if untreated, progress to invasive cancer [3]. Despite advancements in preventive measures, such as HPV vaccines and screening methods like Pap smears, cervical cancer remains a significant public health concern, especially in low-resource settings where access to screening and healthcare services is limited [4–6].

In Rwanda, cervical cancer is the most commonly diagnosed cancer among women, with an incidence rate of 42 per 100,000 women annually [7]. Limited infrastructure and shortages of skilled healthcare providers exacerbate the challenge, leading to delays in diagnosis and treatment [8–10]. Additionally, traditional diagnostic methods such as Pap smears and biopsy tests are labor-intensive, prone to human error, and often inaccessible to rural populations [11, 12]. These limitations underscore the need for innovative and scalable diagnostic solutions.

Recent advancements in artificial intelligence (AI), particularly deep learning (DL), have shown immense promise in automating medical image analysis, improving diagnostic accuracy, and reducing interobserver variability [13–15]. DL models, such as convolutional neural networks (CNNs), have demonstrated their ability to accurately classify cervical cancer images, outperforming traditional machine learning approaches [16–18]. CNN-based models have successfully classified breast, prostate, and colorectal histology, achieved expert-level agreement and provided robust inference on large-scale clinical datasets [19–21]. However, the performance of such models is not much validated in low-resource environments. Most studies rely on curated datasets like The Cancer Genome Atlas (TCGA) and NCT-CRC-HE-100 K produced using standardized staining, uniform hardware, and high-volume pathology laboratories [19], leaving a gap in understanding how these technologies can be adapted for low-resource settings like Rwanda [22, 23].

In addition, emerging computational pathology research has extended AI applications toward clinically meaningful prediction and annotation-efficient learning strategies. For example, DL systems have been developed to predict distant and lymph node metastasis directly from primary tumor histopathology using slide-level labels, while weakly supervised frameworks leverage multi-instance learning and contrastive modeling to enable segmentation using limited annotations [24–26]. These advances demonstrate the growing potential of AI to address complex pathological tasks, but their performance in low-resource environments remains underexplored.

A fundamental limitation of existing cervical cancer AI studies is their treatment of disease as a binary classification problem (normal vs. abnormal). Clinically, cervical carcinogenesis follows a spectrum: normal epithelium progresses through cervical intraepithelial neoplasia (CIN I-III) toward invasive malignancy, with distinct morphological and architectural features at each stage [27]. Rare subtypes such as adenosquamous carcinoma diagnostically relevant in East African populations are underrepresented in available datasets and systematically misclassified by DL models, creating structural bias [28]. While our dataset captures this full pathological spectrum, this study focuses exclusively on the clinically actionable binary task (normal vs. abnormal) to ensure statistical robustness given class imbalance and limited subtype counts.

This study addresses these gaps by evaluating three DL models; ResNet50, EfficientNetB0, and DenseNet121 on a real-world cervical histopathology dataset collected from the University Teaching Hospital of Kigali (CHUK). Our work is framed as feasibility study for binary classification rather than a multi-class diagnostic system. Rather than presenting a deployable diagnostic system, we report feasibility and failure models relevant to clinical adoption in low-resource environments.

## Methods

### Study setting and ethics

This retrospective study analyzed all histopathological images of the cervix from 2018 to 2024 obtained at CHUK, Rwanda’s largest tertiary referral hospital. Each histopathological image included in this study corresponds to a single diagnostic biopsy slide from a unique patient, with no repeated slides or multiple images per individual. Ethical approval was granted by the University of Rwanda, College of Medicine and Health Sciences Institutional Review Board (CMHS IRB) with approval reference CMHS/IRB/341/2024. Clearance was also obtained from the University Teaching Hospital of Kigali (CHUK) Research Ethics Committee under reference number EC/CHUK/146/2024.

### Study population

The target population included patients diagnosed with cervical cancer, those with precancerous changes or with inflammatory lesions, or with normal findings, whose histopathological images are available for analysis. The final analytical cohort therefore consisted of 885 unique patients, each contributing exactly one biopsy slide to the dataset.

### Histopathology workflow and image acquisition

Biopsies were formalin-fixed and paraffin-embedded (FFPE), sectioned at 3–5 $\:\mu\:m$, and stained with hematoxylin and eosin (H&E) according to routing CHUK laboratory protocols. Whole-slide images (WSIs) were digitized at 200x magnification (0.5 $\:\mu\:m/pixel$), providing subcellular resolution appropriate for morphological differentiation. For each WSI, a single diagnostically representative region was selected by certified pathologists; no slide was subdivided into multiple analytical patches. Two certified pathologists independently annotated regions of interest (ROIs) corresponding to representative cellular structures, excluding necrotic, hemorrhagic, out-of-focus, and over-stained areas. Discrepant cases were reviewed in consensus, and ambiguous slides were excluded. This ensured ground truth accuracy and reduced technical noise introduced by histological artefacts and preparation variability.

### Diagnostic taxonomy

The dataset comprised 885 ROI patches organized according to the biological progression of cervical neoplasia. Each ROI corresponds to a single biopsy slide from one patient, such that the number of images, slides, and patients is identical (*n* = 885). Normal tissues included columnar epithelium, squamocolumnar junction, and mature squamous epithelium. Precancerous lesions were stratified into low, moderate, and high-grade intraepithelial neoplasia, and malignant neoplasms included carinoma in situ, invasive adenocrarcinoma, adenosquamous carcinoma, and invasive squamous carcinoma.

This taxonomy aligns with WHO histopathology guidelines and avoids conflating dysplasia grades with invasive carcinoma, an error common in earlier works that obscures clinically meaningful diagnostic boundaries [12].

### Image preprocessing

Images were resized to 224 × 224 pixels and normalized to CNN input requirements. All images were originally acquired as color H&E-stained slides. Grayscale images were ensured to be in RGB format to maintain compatibility with the CNN input requirements.

To mitigate class imbalance without distorting intra-cellular morphology, class weighted loss and domain consistent augmentations were used, including rotation ($\:<{15}^{0})$, mirror flips, and controlled brightness/contrast adjustments, which preserve cytological structure while increasing variability [29]. Class weights were computed using an inverse-frequency weighting strategy derived from the distribution of classes in the training set. Under this approach, class with fewer samples were assigned proportionally higher weights during optimization to compensate for underrepresentation.

### Deep learning architectures

Three pretrained CNN architectures were evaluated: ResNet50, EfficientNetB0, and DenseNet121. These architectures were chosen due to their proven reliability in medical image classification and their suitability for low-resource computational environments. Residual connectivity in ResNet50 facilitates gradient propagation; compound scaling in EfficientNetBO balances depth and width with minimal parameter growth; and DenseNet121 enables feature reuse across layers, improving parameter efficiency [30, 31]. All models were initialized using ImageNet weights to leverage transfer learning for morphological feature extraction.

**Fig. 1:**
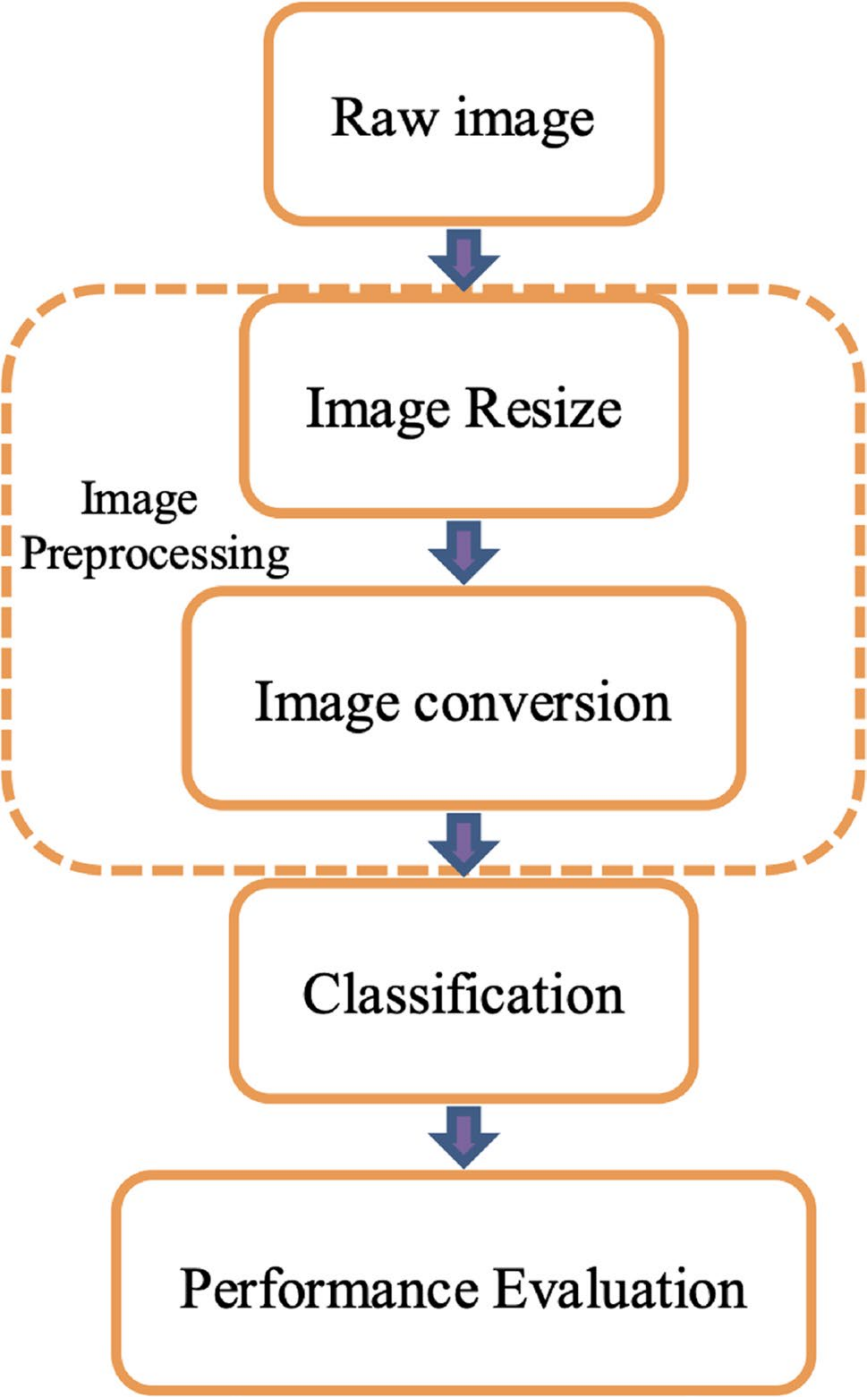
Block diagram of cervical cancer images classification task

Figure 1 shows how raw histopathology images undergo standardized preprocessing before being propagated through the deep learning architecture for inference.

### Training strategy and leakage prevention

To prevent patient-level leakage, all dataset partitions were performed at the case level. Multiple ROI patches from the same patient were never distributed across training, validation, and testing subsets, a methodological flaw known to inflate DL results in digital pathology [32, 33].

Data were split into training (70%), validation (20%), and test (10%) partitions using stratified sampling to preserve class distribution. Models were trained using Adam optimizer (learning rate: 0.001), batch size 32, cosine learning rate decay, and early stopping (patience: 10 epochs) [34].

### Evaluation and statistical analysis

The models were evaluated using several metrics, including accuracy, sensitivity, specificity, precision, F1-score, and ROC-AUC. All reported performance metrics reflect generalization across patients rather than within patient memorization of staining or sectioning artifacts. Accuracy was calculated as the proportion of correctly classified instances over the total number of instances, while sensitivity measured the ability to identify positive cases. Specificity assessed the model’s capability to correctly classify negative cases, and precision evaluated the proportion of true positives among all positive predictions. The F1 score, a harmonic mean of precision and recall, offered a balanced assessment. The Receiver Operating Characteristic (ROC) curve was plotted, and the Area Under the Curve (AUC) was calculated to summarize the trade-off between sensitivity and specificity. Table 1 presents the confusion matrix used for evaluating the binary classification task. Moreover, 95% (95%) confidence intervals were estimated via nonparametric bootstrapping (1,000 resamples). Discriminative performance was compared using the Delong test for AUC differences, and paired misclassification rates were examined using McNemar’s test with Holm-Bonferroni correction. To assess probabilistic reliability, calibration curves and Brier scores were computed [35, 36].

**Table 1:** Confusion matrix for binary classification

		Predicted category	
		Positive category – Normal (0)	Negative category – Abnormal (1)
Actual category	Positive category –Normal (0)	True Positive (TP)	False Negative (FN)
	Negative category – Abnormal (1)	False Positive (FP)	True Negative (TN)

### Dataset description

The dataset comprised 885 histopathology image driven from 885 distinct biopsy slides, each corresponding to a single unique patient. All image patches obtained from digitized hematoxylin-and-eosin-stained sections at the University Teaching Hospital of Kigali (CHUK). Thus, the dataset represents 885 patients, with one representative region-of-interest (ROI) extracted per slide, ensuring a one-to-one correspondence between image, slide, and patient. This design eliminates intra-patient redundancy and mitigates the risk of patient-level information leakage during model development and evaluation.

For analytical clarity and to maintain clinical relevance, the dataset was structured into three main diagnostic groups: normal tissues, precancerous lesions, and malignant neoplasms. Within these groups, ten distinct histopathological classes were represented. This structure reflects the pathological continuum of cervical carcinogenesis and prevents overlap between intraepithelial dysplasia and invasive cancers. Three classes in normal tissues kept separate because they exhibit characteristic cytological morphology that informs both clinical assessment and automated classification models. Precancerous lesions were stratified using the cervical intraepithelial neoplasia (CIN) framework. This CIN spectrum represents progressive epithelial dysplasia associated with HPV-driven oncogenesis and is distinguishable from normal tissue by nuclear pleomorphism, increased mitotic figures, and architectural disorganization. The third group, malignant neoplasms included four classes. Carcinoma in situ reflects full-thickness dysplasia without stromal invasion, whereas the invasive subtypes demonstrate basement membrane disruption, stromal infiltration, and destructive growth patterns.

**Fig. 2:**
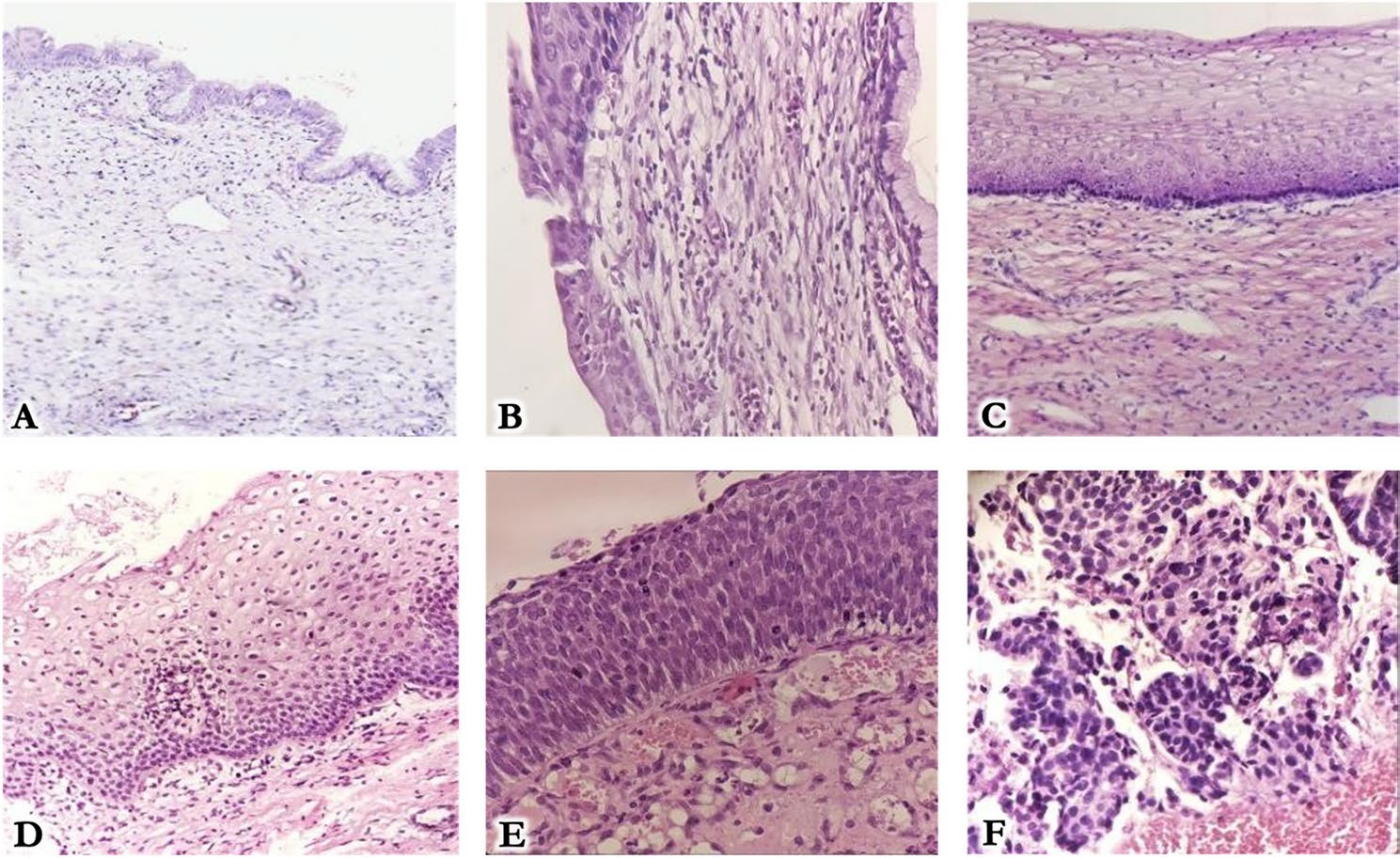
Representative histopathological samples illustrating the morphological. **A** Category 1 – normal columnar epithelium (H&E stain, X200 magnification); **B** Category 2 – normal squamo-columnar junction (H&E stain, X200 magnification); **C** Category 3 – normal squamous epithelium (H&E stain, X200 magnification); **D** Category 4 – abnormal low grade intraepithelial neoplasm (H&E stain, X200 magnification); **E **Category 5 – abnormal high grade squamous intraepithelial neoplasm (H&E stain, X200 magnification; **F** Category 6 – abnormal invasive carcinoma (H&E stain, X200 magnification)

Table 2 shows the complete distribution of classes across the three diagnostic groups. Representative examples of selected categories are shown in Fig. 2 to visually illustrate morphological variation across the neoplasia continuum.

**Table 2:** Distribution of histopathological categories in the dataset

Diagnostic group	Histopathological class	Frequency
Normal tissue		396
	Columnar epithelium	208
	Squamocolumnar junction	62
	Squamous epithelium	126
Abnormal:		489
Precancerous lesions (CIN spectrum)	Low-grade intraepithelial neoplasm	64
	Moderate-grade intraepithelial neoplasm	39
	High-grade intraepithelial neoplasm	59
Malignant neoplasms	Carcinoma in situ	64
	Invasive adenocarcinoma	62
	Adenosquamous carcinoma	23
	Invasive squamous carcinoma	178
Total		885

### Statistical and computational framework

The deep learning models were implemented in Python using TensorFlow and Keras libraries, with Scikit-learn used for statistical evaluations. ROC curves were generated for binary classification, and AUC values were calculated to assess the overall performance of the models. Confidence intervals for accuracy, sensitivity, specificity, and AUC were estimated using bootstrapping techniques. Statistical significance was assessed by comparing the performance metrics across the three models.

## Results

### Binary classification performance

The three CNN models achieved high performance in the binary classification of cervical histopathology images into normal and abnormal categories, with differences in error patterns across architectures. On the held-out test set, EfficientNetB0 obtained the highest point accuracy of 0.99 (95% CI 0.97-1.00), with sensitivity 0.98 (0.94-1.00), specificity 1.00 (1.00–1.00), F1-score 0.99 (0.97-1.00), ROC-AUC 0.99 (0.98-1.00), and the lowest Brier score (0.02) (Table 3). ResNet50 achieved an accuracy of 0.91 (0.84–0.97), sensitivity 0.94 (0.87-1.00), specificity 0.87 (0.75–0.97), F1-score 0.92 (0.86–0.97), ROC-AUC 0.96 (0.91–0.99), and Brier score of 0.07. DenseNet121 yielded an accuracy of 0.86 (0.79–0.93), sensitivity of 0.86 (0.76–0.96), specificity 0.86 (0.74–0.97), F1-score 0.88 (0.80–0.94), ROC-AUC 0.96 (0.92–0.99), and the highest Brier score (0.08). These results indicate that, while all three models are capable of distinguishing normal from abnormal tissue, EfficientNetB0 offers the best calibrated performance, with ResNet50 also performing strongly and DenseNet121 trailing behind.

**Fig. 3:**
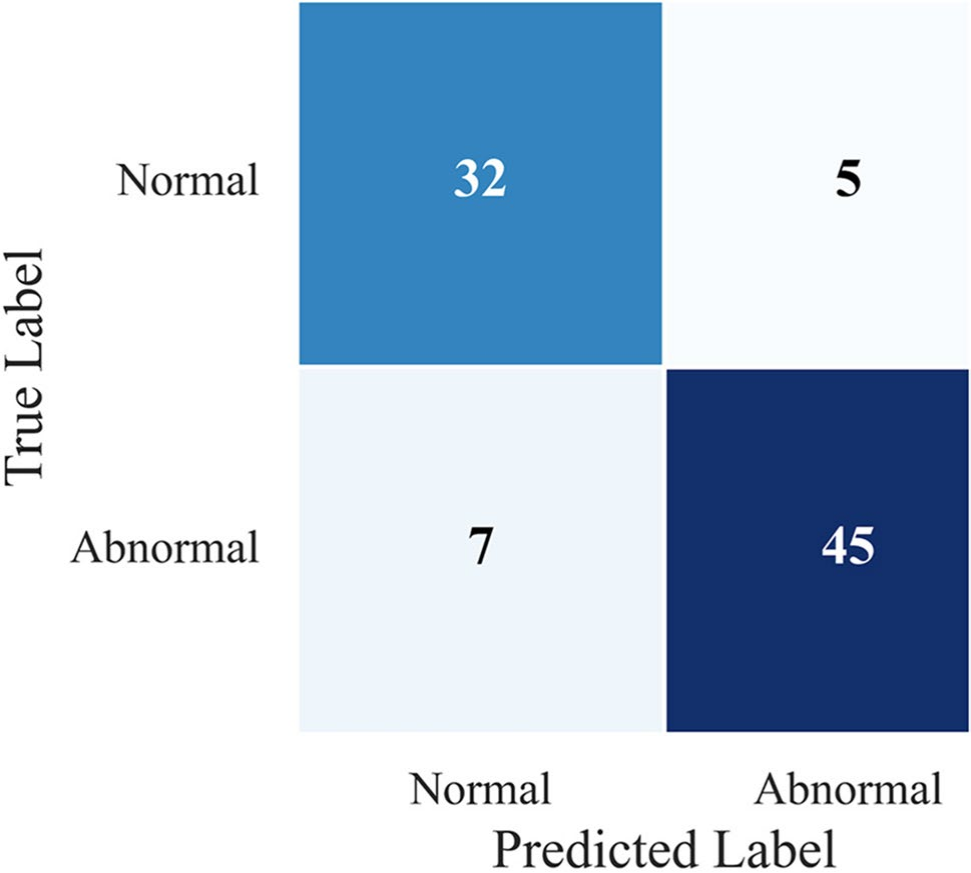
Confusion matrix for DenseNet121 model

**Fig. 4:**
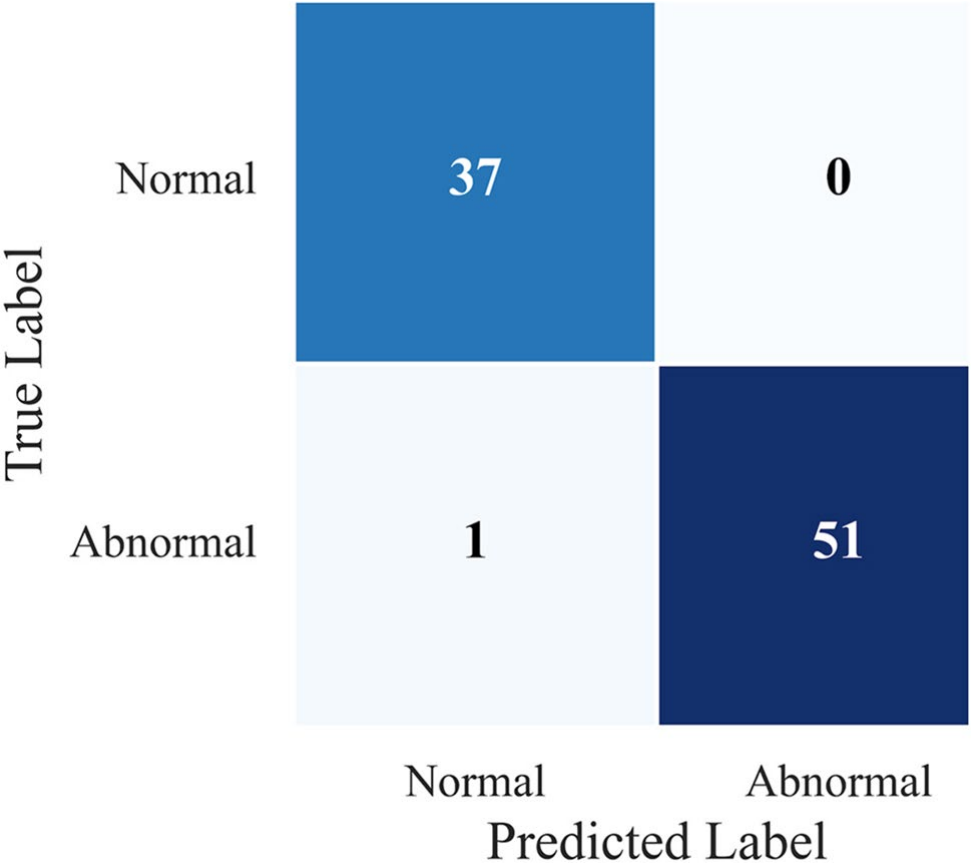
Confusion matrix for EfficientNetB0 model

**Fig. 5:**
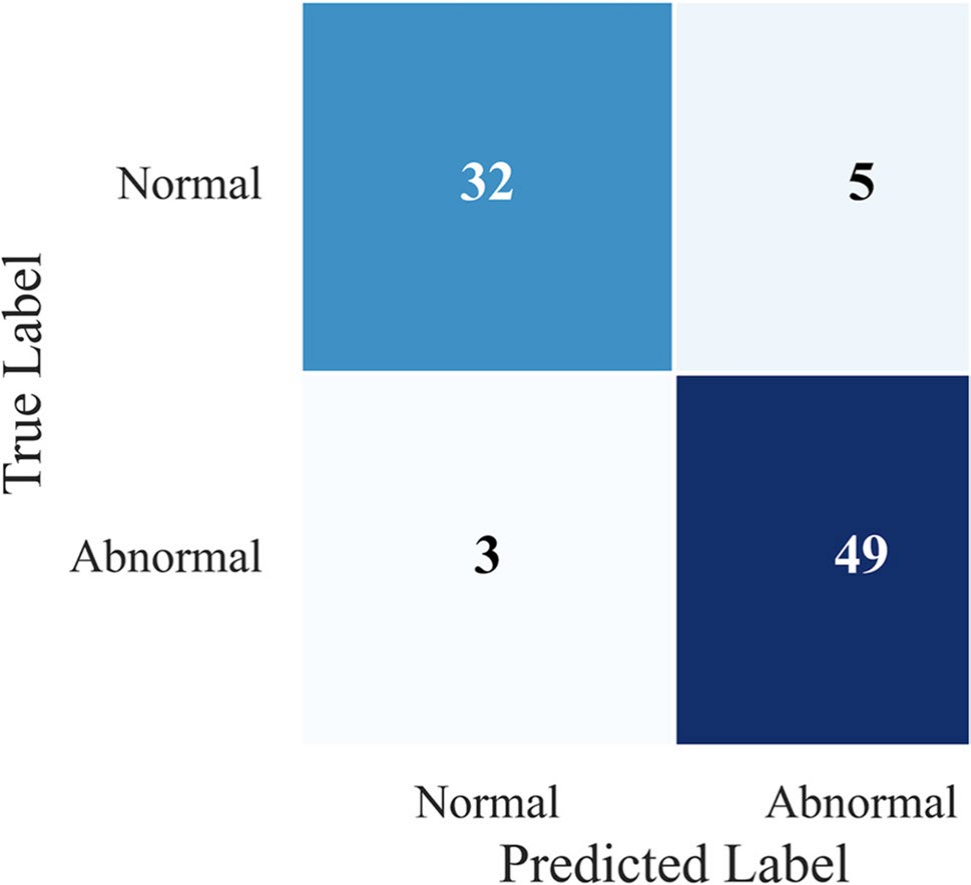
Confusion matrix for ResNet50 model

The confusion matrices in Figs. 3, 4 and 5 provide a clear characterization of individual model error patterns based on the held-out binary test subset of 89 images, which represents the stratified evaluation portion of the full dataset of 885 histopathology samples. DenseNet121 correctly classified 32 of 37 normal images as normal and 45 of 52 abnormal images as abnormal, while misclassifying 5 normal samples as abnormal (false positives) and 7 abnormal samples as normal (false negatives), indicating limited sensitivity to malignant architectural features. In contrast, EfficientNetB0 demonstrated a more robust discriminatory profile, correctly identifying all 37 normal samples and 51 of 52 abnormal samples, yielding zero false positives and only one false negative, which explains its perfect specificity and near perfect sensitivity. ResNet50 exhibited balanced performance, correctly classifying 32 of 37 normal samples and 49 of 52 abnormal samples, with 5 false positives and 3 false negatives, suggesting a slight trade-off between conservative boundary selection and sensitivity to pathological cues.

### Model performance curves

**Fig. 6:**
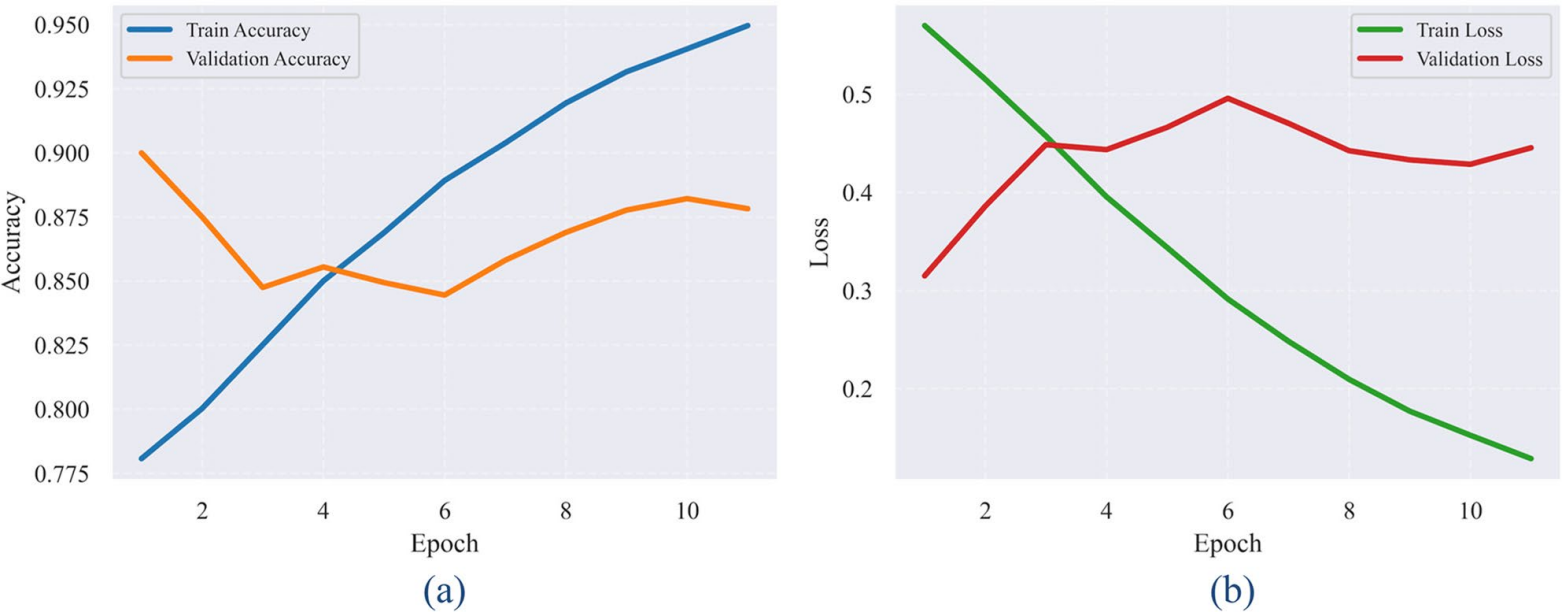
ResNet50 training and validation curves: **a** accuracy; **b** loss

**Fig. 7:**
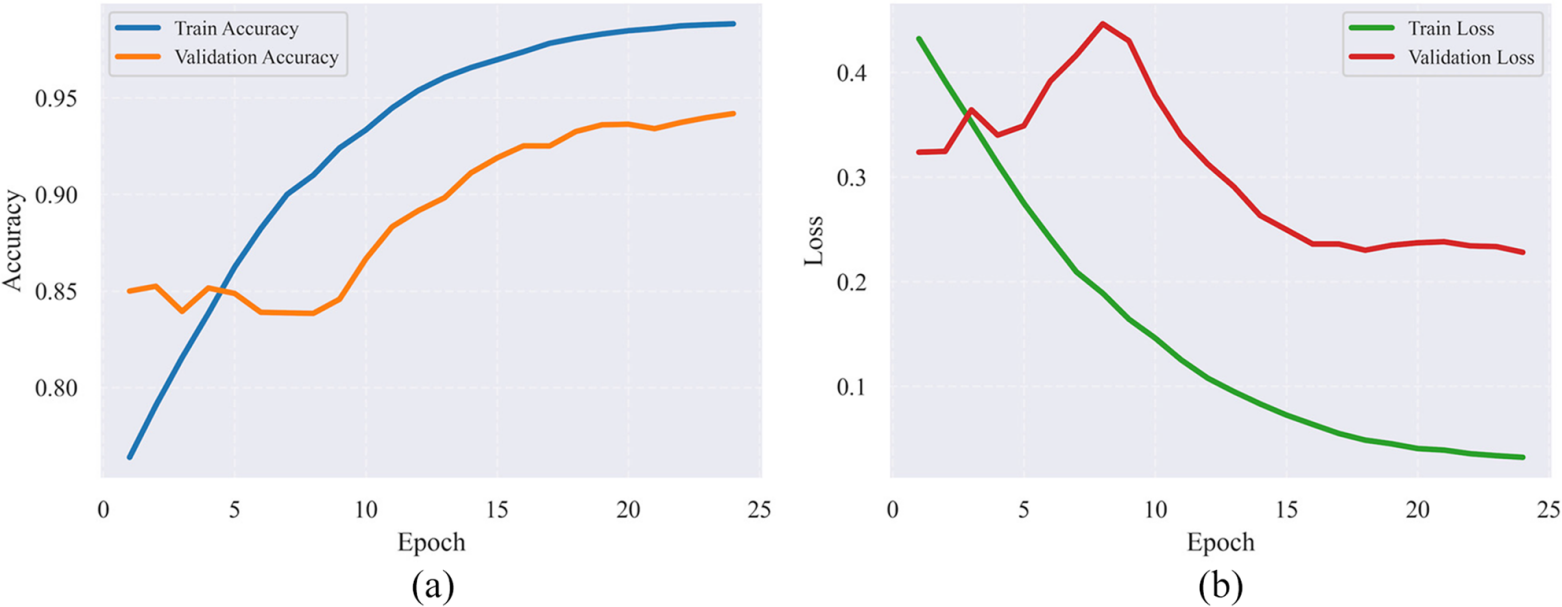
EfficientNetB0 training and validation curves: **a** accuracy; **b** loss

**Fig. 8:**
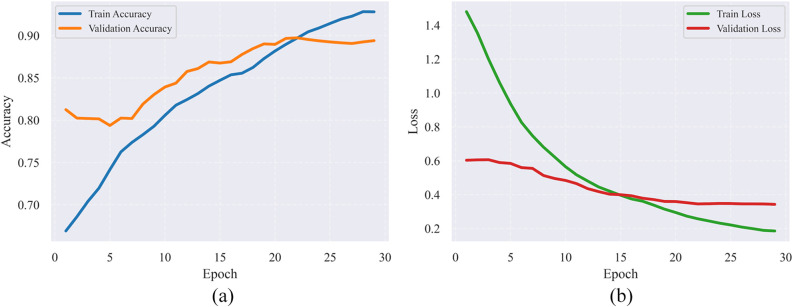
DenseNet121 training and validation curves: **a** accuracy; **b** loss

The training and validation curves provide insight into optimization dynamics and overfitting behavior (Figs. 6, 7 and 8). For ResNet50 (Fig. 6), training and validation accuracy increased steadily with epochs, while the corresponding loss curves declined smoothly and remained closely aligned, indicating stable learning and limited overfitting. EfficientNetB0 (Fig. 7) showed similar trends, with both training and validation accuracy approaching 1.0 by the end of training; modest oscillations in validation accuracy reflect the small size of the test set rather than systematic instability. In contrast, DenseNet121 (Fig. 8) exhibited a wider gap between training and validation performance: training accuracy continued to rise, whereas validation accuracy plateaued earlier and fluctuated, suggesting a tendency toward overfitting and reduced generalization capacity in this relatively small dataset.

### Calibration and ROC analysis

**Fig. 9:**
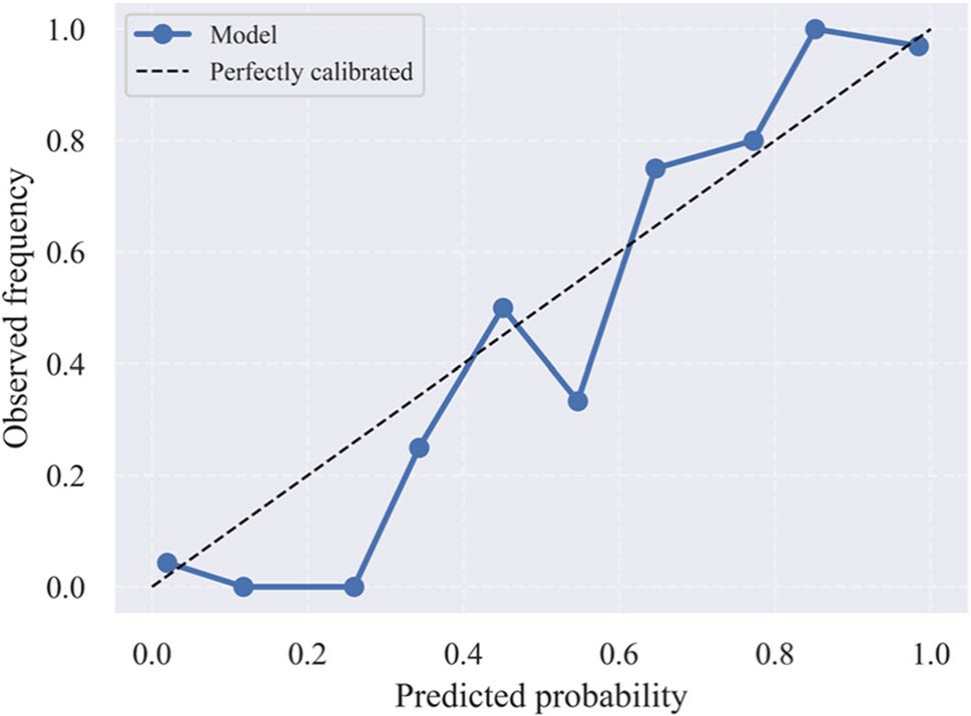
ResNet50 calibration curve

**Fig. 10:**
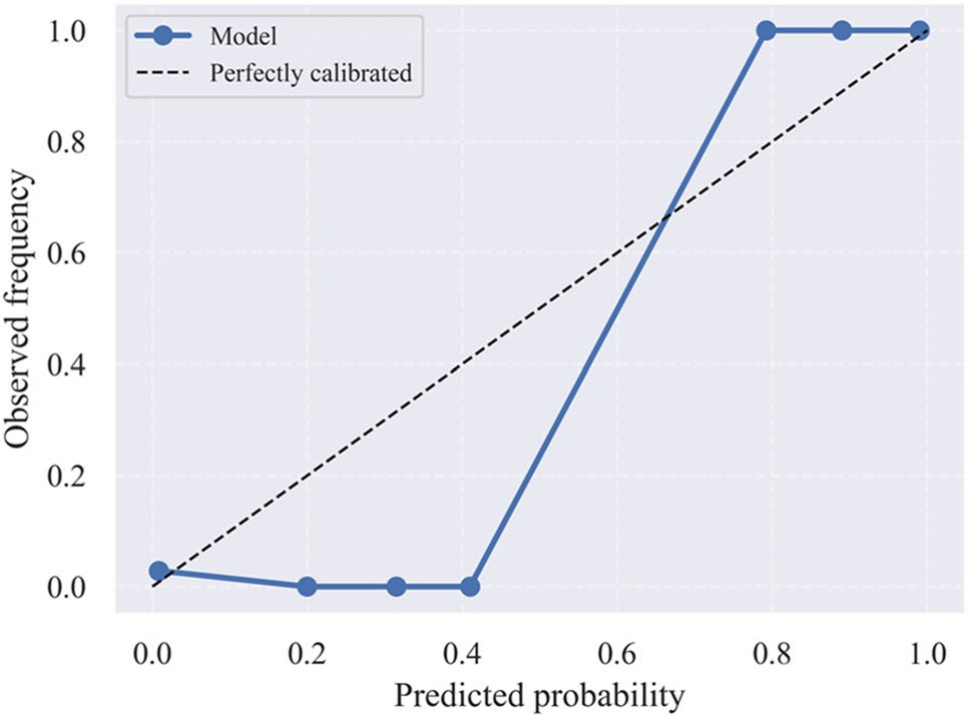
EfficientNetB0 calibration curve

**Fig. 11:**
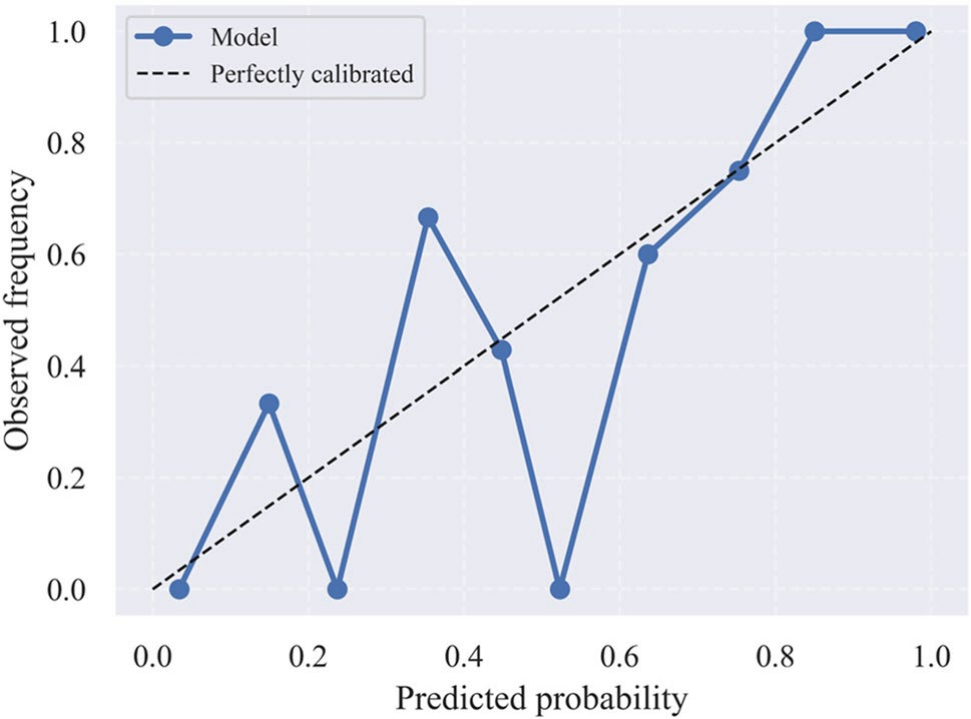
DenseNet121 calibration curve

Calibration plots (Figs. 9, 10 and 11) align with the Brier scores reported in Table 3. EfficientNetB0 showed the best calibration, with predicted probabilities lying close to the diagonal “perfect calibration” line across most probability bins, consistent with its low Brier score (0.02). ResNet50 displayed wider deviations around the diagonal, particularly in intermediate probability ranges, reflecting moderate over or under estimation of risk in some bins and a higher Brier score (0.07). DenseNet121 deviated most from the ideal line and produced overconfident predictions at mid-range probabilities, in agreement with its Brier score of 0.08 and lower accuracy.

### Receiver operating characteristic (ROC) analysis

**Fig. 12:**
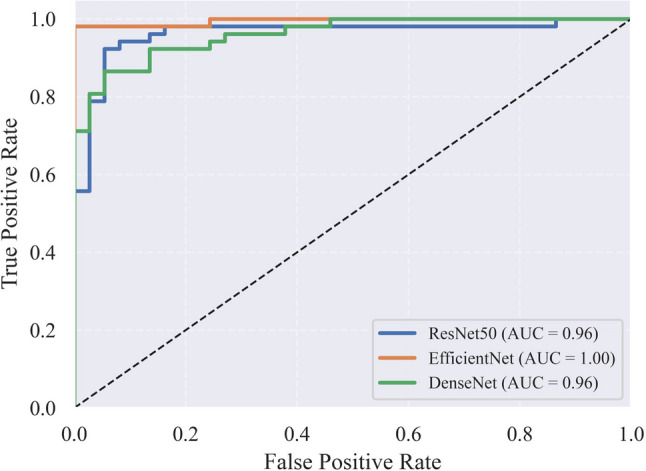
Receiver operating characteristic curve

ROC curves of the three models are represented in Fig. 12. EfficientNetB0 achieved the highest discrimination, with an ROC-AUC of 0.99, followed by ResNet50 and DenseNet121, each with an AUC of 0.96 (Table 3). The ROC curve for EfficientNetB0 rises toward the upper left corner, indicating that high sensitivity can be achieved with almost no increase in false-positive rate. The ResNet50 and DenseNet121 curves also demonstrate excellent overall discrimination, but with lower sensitivity at very low false-positive rates compared with EfficientNetB0. These results confirm that all models operate in a high-AUC regime, with EfficientNetB0 providing the best overall trade-off between sensitivity and specificity.

### Classification performance of all three models

Table 3 summarizes the performance metrics for all models across binary classification tasks. EfficientNetB0 outperformed the other models, with high accuracy, sensitivity, and specificity in binary classification.

**Table 3:** Performance results of all models

Model	Accuracy	Preci-sion	Recall (Sensitivity)	Specificity	F1-score	ROC AUC	Brier score
ResNet50	0.91 (0.84–0.97)	0.91	0.94 (0.87–1.0)	0.865 (0.75–0.97)	0.92 (0.86–0.97)	0.96 (0.91–0.99)	0.07
EfficientNetBO	0.99 (0.97–1.0)	1.0	0.98 (0.94–1.0)	1.0 (1.0–1.0)	0.99 (0.97–1.0)	0.99 (0.98–1.0)	0.02
DenseNet121	0.86 (0.79–0.93)	0.9	0.86 (0.76–0.96)	0.86 (0.74–0.97)	0.88 (0.80–0.94)	0.96 (0.92–0.99)	0.08

## Discussion

The findings of this study demonstrate that convolutional neural networks can achieve high diagnostic accuracy for automated binary classification of cervical histopathology images in a resource-limited setting. Using 885 patient level images and a stratified test subset of 89 samples, EfficientNetB0 achieved the strongest performance, accuracy 0.99 (95% CI 0.97-1.00), and the lowest Brier score (0.02). These results indicate that compact, computationally efficient architectures can distinguish normal from abnormal epithelium, offering a clinically relevant pathway for augmenting pathology services in settings with limited specialist capacity [1, 17, 18].

ResNet50 and DenseNet121 also demonstrated high discrimination, though with differences in calibration and error characteristics. ResNet50 achieved accuracy of 0.91 (0.84–0.97) and ROC-AUC 0.96 (0.91–0.99), representing a strong and balanced model suitable for environments where ResNet-based tooling and interpretability are already established. DenseNet121, despite a similar AUC (0.96 [0.92–0.99]), showed reduced reliability with a higher Brier score (0.08), and its optimization patterns indicated greater susceptibility to overfitting likely a consequence of its dense connectivity and the modest dataset size.

The confusion matrix findings reinforce these distinctions. EfficientNetB0 produced the most favorable error profile, with zero false positives and only one false negative, demonstrating excellent sensitivity to malignant features while avoiding unnecessary over calling. ResNet50 misclassified slightly more samples but maintained strong balance between sensitivity and specificity. DenseNet121 incurred the highest number of false negatives, highlighting its reduced robustness in detecting subtle morphological abnormalities. In a clinical triage context where failure to detect abnormal tissue is far more consequential than false alarms, EffecientNetB0’s operating characteristics represent a pragmatic advantage.

The study’s results underscore the need for further refinements in dataset preparation and model optimization. Therefore, addressing class imbalance could significantly enhance minority class recognition [37, 38]. Moreover, integrating interpretability techniques, such as saliency maps or attention mechanisms, could provide clinicians with more transparent insights into model predictions, fostering greater trust in DL- based systems [39].

Despite the promising outcomes, the study has limitations that must be acknowledged. The dataset, although representative of Rwanda’s population, may not generalize to other regions with differing demographic and pathological profiles. The relatively small sample size, particularly for minority classes, constrained the models’ ability to learn nuanced features. Expanding the dataset with images from diverse sources and incorporating multi-modal data, such as clinical and demographic information, could further enhance the models’ robustness and applicability.

## Conclusion

This study provides evidence that deep learning models, particularly EfficientNetB0 can achieve high diagnostic accuracy, good calibration, and robust generalization for binary classification of cervical histopathology images in a resource constrained clinical environment. Using patient level stratification and rigorous evaluation, EfficientNetB0 demonstrated near perfect discrimination between normal and abnormal cervical epithelium, outperforming ResNet50 and DenseNet121 in both classification performance and reliability of predicted probabilities. These findings are consistent with recent literature showing the strong performance of compact CNN architectures in digital pathology workflows, and reinforce the growth consensus that AI-assisted microscopy can meaningfully support early detection and triage in settings where specialist pathology expertise remains limited.

Nonetheless, the study’s single center design, modest dataset size, and absence of external validation highlight the continued need for multi-institutional datasets, harmonized imaging protocols, and prospective evaluation before such systems can be deployed clinically. Future research should prioritize cross-domain generalization, calibration monitoring, hierarchical clinical decision integration, and explainability tools such as attention heatmaps and fine-grained feature attribution to strengthen clinician trust and regulatory readiness. Within these constraints, our findings support the feasibility of deploying lightweight deep learning models as adjunctive diagnostic tools in low- and middle-income settings, contributing to broader global health efforts aimed at reducing cervical cancer morbidity and mortality through improved diagnostic capacity.

## Data Availability

The data utilized in this study were acquired from CHUK and were managed in compliance with ethical guidelines and data protection laws. Due to these regulations, the datasets are not publicly accessible. However, they can be made available upon reasonable request to the corresponding author, subject to approval from the University of Rwanda Ethical Committee and the CHUK.

## Abbreviations

ACE-DS: African Centre of Excellence in Data Science
AI: Artificial Intelligence
AIMS: African Institute for Mathematical Sciences
AUC: Area Under the Curve
CHUK: University Teaching Hospital of Kigali (Centre Hospitalier Universitaire de Kigali)
CMHS IRB: College of Medicine and Health Sciences Institutional Review Board
CI: Confidence Interval
CIN: Cervical Intraepithelial Neoplasia
CNN: Convolutional Neural Network
CRC: Colorectal Cancer
DL: Deep Learning
FFPE: Formalin-Fixed, Paraffin-Embedded
FN: False Negative
FP: False Positive
GAN: Generative Adversarial Network
H&E: Hematoxylin and Eosin
HPV: Human Papillomavirus
NCT: National Center for Tumor Diseases
NIH: National Institute of Health
RGB: Red, Green, Blue
ROC: Receiver Operating Characteristic
ROC-AUC: Receiver Operating Characteristic Area Under the Curve
ROI: Region of Interest
TN: True Negative
TP: True Positive
TCGA: The Cancer Genome Atlas
WSI: Whole-Slide Image
WHO: World Health Organization
